# Revealing the formation mechanism and band gap tuning of Sb_2_S_3_ nanoparticles

**DOI:** 10.3762/bjnano.12.76

**Published:** 2021-09-10

**Authors:** Maximilian Joschko, Franck Yvan Fotue Wafo, Christina Malsi, Danilo Kisić, Ivana Validžić, Christina Graf

**Affiliations:** 1 Hochschule Darmstadt - University of Applied Sciences, Fachbereich Chemie- und Biotechnologie, Stephanstr. 7, D-64295 Darmstadt, Germany; 2 Vinča Institute of Nuclear Sciences, National Institute of the Republic of Serbia, University of Belgrade, Mike Petrovića Alasa 12-14, 11351 P.O. Box 522, Belgrade, Serbia

**Keywords:** band gap, kinetics, nanoparticles, Sb_2_S_3_, solar cells

## Abstract

Sb_2_S_3_ is a promising nanomaterial for application in solar cells and in the fields of electronics and optoelectronics. Herein, Sb_2_S_3_ nanoparticles were prepared via the hot-injection approach. In contrast to earlier work, the reaction temperature was decreased to 150 °C so that the reaction was slowed down and could be stopped at defined reaction stages. Thereby, the formation mechanism of the nanomaterial and the associated kinetics could be revealed. Based on morphological and structural analyses, it is suggested that seed particles (type 0) formed immediately after injecting the antimony precursor into the sulfur precursor. These seeds fused to form amorphous nanoparticles (type I) that contained a lower percentage of sulfur than that corresponding to the expected stoichiometric ratio of Sb_2_S_3_. The reason for this possibly lies in the formation of an oxygen- or carbon-containing intermediate during the seeding process. Afterward, the type I nanoparticles aggregated into larger amorphous nanoparticles (type II) in a second hierarchical assembly process and formed superordinate structures (type III). This process was followed by the crystallization of these particles and a layer-like growth of the crystalline particles by an Ostwald ripening process at the expense of the amorphous particles. It was demonstrated that the kinetic control of the reaction allowed tuning of the optical band gap of the amorphous nanoparticles in the range of 2.2–2.0 eV. On the contrary, the optical band gap of the crystalline particles decreased to a value of 1.7 eV and remained constant when the reaction progressed. Based on the proposed formation mechanism, future syntheses for Sb_2_S_3_ particles can be developed, allowing tuning of the particle properties in a broad range. In this way, the selective use of this material in a wide range of applications will become possible.

## Introduction

The search for efficient, renewable energies with broad availability has become one of the most important challenges of our century. With a usable radiation energy per year that is several times larger than the energy consumption of the world [1], solar energy is a suitable source for future energy supply. However, there are several requirements for materials to be eligible for application in the field of photovoltaics, such as high absorption performance, nontoxicity, abundance, efficiency, and low cost.

As a semiconductor with a low band gap and a high absorption coefficient, antimony(III) sulfide (Sb_2_S_3_) has become a promising absorption material for photovoltaic applications [2–4]. Furthermore, the material is also suitable for various electronic and optoelectronic applications, such as energy storage [5] or optical data storage [6]. Sb_2_S_3_ appears in two forms: an orange, amorphous form and a grayish-black, crystalline form, known as the mineral stibnite [7–8].

Sb_2_S_3_ nanomaterials with a diverse morphology and a broad distribution of band gap values were synthesized by solvothermal [9], hydrothermal [10], and sonochemical [11] approaches, as well as by chemical bath [12] and chemical vapor deposition [13] methods. Up to now, the syntheses of Sb_2_S_3_ nanomaterials lack sufficient control of the growth conditions. The result are nanoparticles of which the size, shape, and crystallinity can only be tuned to a limited extent. However, for several applications, such as electronic circuits [14], it is crucial to adjust these parameters. Also, when aiming for continuous production by transferring the synthesis into a microreactor, it is necessary to know and control synthesis and particle parameters in a broader range [15]. Mainly two synthesis strategies to gain nanoparticles with uniform size and shape have been described in the literature in the past two decades: the heat-up and the hot-injection methods [16]. While the former is rarely applied to synthesize Sb_2_S_3_ nanoparticles [9], the hot-injection approach has been used in several studies [17–19].

Syntheses reported so far, using the hot-injection method at a temperature between 180 and 240 °C [17–18,20], yield nanoparticles not smaller than 100 nm or, almost instantaneously, rods, tubes, or wires in the micron size.

Abulikemu et al. have investigated the influence of different sulfur and antimony precursors, injection (140–220 °C) and reaction temperature (100–220 °C), and the overall reaction time (90 s to 2.5 h) on the structural, optical, and morphological properties of Sb_2_S_3_ nanoparticles via the hot-injection route [19]. They have shown that a higher injection temperature leads to smaller nuclei and a higher reaction temperature leads to larger particles. Furthermore, they have concluded that a chlorine-containing antimony precursor affects the morphology and crystallinity of the particles. Nevertheless, the study of Abulikemu et al. focused on using bis(trimethylsilyl) sulfide (TMS) as sulfur precursor since with this compound, the highest reactivity was reached. However, TMS is toxic and also costly compared to the widely used elemental sulfur, limiting a broad use in the preparation of Sb_2_S_3_ materials [19].

Li et al. have performed mechanistic studies on the temperature dependency of Sb_2_S_3_ nanoparticles in the range of 180–210 °C to facilitate the synthesis process following the hot-injection method with a sulfur–oleylamine (S-OlAm) precursor. They have found that the temperature influences the crystallinity, shape, and size of the particles [21].

These studies revealed growth processes comprising a primary formation of amorphous Sb_2_S_3_ nanoparticles, which started to crystallize in an orthorhombic structure and continued to grow. However, in the studies performed so far, both the formation of the nanoparticles and the subsequent growth occurred rapidly. Detailed knowledge of the exact formation and growth mechanisms, and in particular the associated kinetics of Sb_2_S_3_ nanoparticles, is therefore still lacking. Nevertheless, it is necessary to understand the nanomaterial formation mechanism to achieve control over the morphological and optical properties of the particles, which is crucial for the further application.

In the present work, a hot-injection approach at a moderate temperature (150 °C) is presented. The reaction was analyzed in the time sections from 30 s to 30 h. The precursor S-OlAm was selected to achieve a high reactivity while avoiding toxic substances such as TMS. A relatively low injection temperature was chosen to slow down the reaction rate, and hence to increase the duration of different reaction steps and decrease the primary particle size. Systematically, the Sb_2_S_3_ nanoparticles in the different formation steps were analyzed regarding morphology, crystallinity, and optical properties, and a detailed proposal for a formation mechanism was elaborated. The mechanism involves the seeding process, growth of amorphous particles, crystallization of the particles, and the following growth of the crystals. The synthesis demonstrated that it is possible to tune the band gap of the Sb_2_S_3_ nanoparticles until the particles reach a fully crystalline state.

## Results and Discussion

The Sb_2_S_3_ nanoparticles were synthesized via a hot-injection synthesis at 150 °C, where a complex consisting of Sb and 2-ethylhexanoic acid (Sb-EHA) was injected into a S-OlAm precursor solution. Subsequently, the reaction took place, the course of which was characterized by several color changes. Immediately after injecting the precursor, the clear, yellowish reaction mixture turned orange and then red but still stayed clear. After 2 min reaction time, the solution became turbid and changed back to an orange color. Next, the mixture turned red (≈20 min) and brown (≈8 h) until it finally became grayish-black (≈18 h). To follow the formation kinetics of the nanoparticles, the reaction was stopped at different characteristic points in time by abrupt cooling. Afterward, the received products were washed by repeated centrifugation and redispersion and by washing with HAS, a 2:3 volumetric mixture of chlorobenzene and 1,2-dichlorobenzene to remove excess sulfur [22]. The experiments were repeated at least three times to ensure reproducibility.

### Morphology and structure

**Figure 1:**
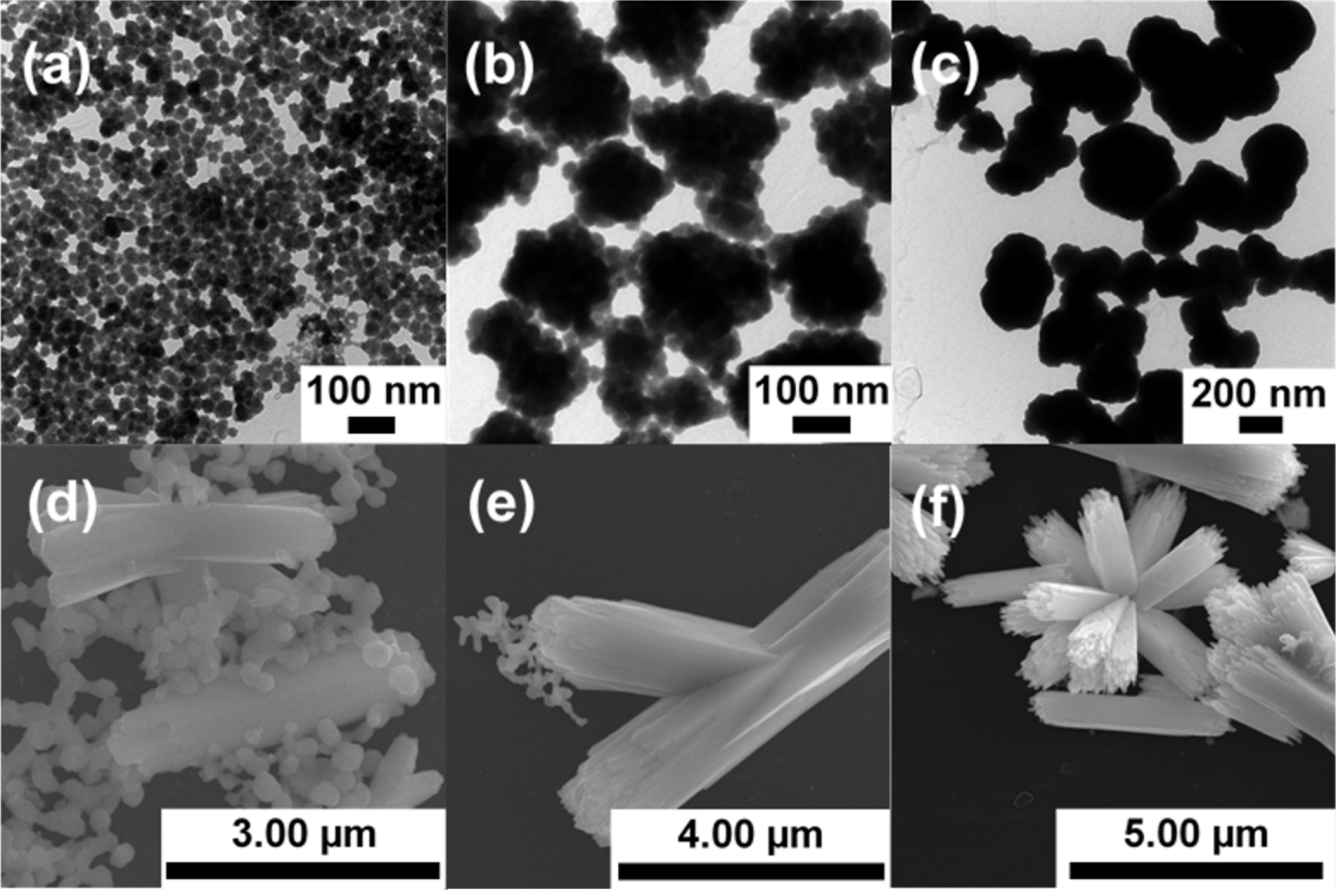
Electron microscopy (EM) images of Sb_2_S_3_ nanoparticles after different reaction times: (a) TEM, 2 min, (b) TEM, 5 min, (c) TEM, 30 min, (d) SEM, 12 h, (e) SEM, 16 h, and (f) SEM, 30 h.

At first, the as-synthesized products obtained after different reaction time points were characterized by transmission electron microscopy (TEM, Figure 1a–c) and scanning electron microscopy (SEM, Figure 1d–f). The images show that small nanoparticles (type I) were formed about 2 min (Figure 1a) after injecting the antimony precursor Sb-EHA. The bright orange product contained nanoparticles with a diameter of 33 ± 5 nm (a summary of the evolution of the particle size throughout the synthesis can be found in Table 1). The nanoparticles were irregularly formed but, in general, spherically shaped.

**Table 1:** Time-dependent characteristics of Sb_2_S_3_.

reaction time	size of amorphous particles/aggregates (nm)	length of crystalline particles (µm)	width of crystalline particles (µm)	Sb/S molar ratio (energy-dispersive X-ray analysis, EDX)	band gap ±0.03 (eV)

2 min	33 ± 5	—	—	48:52	2.18

5 min	210 ± 30	—	—	41:59	2.12

10 min	220 ± 30	—	—	41:59	2.07

30 min	210 ± 30	—	—	38:62	2.07

2 h	230 ± 20	—	—	42:58	2.07

5 h	220 ± 30	—	—	43:57	2.07

8 h	220 ± 30	≈1.0	≈0.22	42:58	2.05/1.72^a^

12 h	220 ± 30	2.6 ± 0.9	0.6 ± 0.2	41:59	2.01/1.68^a^

16 h	150 ± 40	5.7 ± 1.8	1.0 ± 0.3	40:60	—/1.72^b^

18 h	—	5.5 ± 1.9	1.0 ± 0.4	40:60	1.72

30 h	—	5.4 ± 1.6	1.0 ± 0.3	39:61	1.71

^a^The two band gaps corresponded to an amorphous and a crystalline species present in the sample (see main text for details). ^b^The amorphous fraction in this stage was too small to cause a visible slope in the reflectance spectrum.

With increasing reaction time, the dispersion turned darker and became more reddish. After 5 min (Figure 1b), larger clusters (type II) were found, consisting of approximately 20–30 smaller individual nanoparticles. The smaller nanoparticles had the same diameter as the type I nanoparticles, leading to the assumption that the former ones were aggregating. These aggregates tended to have a spherical shape but were somewhat irregularly formed. The mean diameter of the aggregates was 210 ± 30 nm. Neither the appearance nor the size (220 ± 30 nm) of the obtained nanostructures significantly changed when stopping the reaction after 10 min (see Figure S1a in Supporting Information File 1).

As the reaction continued, a dark red dispersion was obtained. While the average particle size (210 ± 30 nm) remained unchanged 30 min after the reaction had been started (Figure 1c), the aggregated type I nanoparticles now appeared to have merged as the type II nanoparticles preserved the shape, but no individual type I nanoparticles could be identified anymore in TEM at this stage. Also, the type II nanoparticles appeared to have formed superordinate structures (type III). Such multistep hierarchical growth mechanisms have already been observed for different materials, such as TiO_2_ [23], ZnO [24], and Co [25]. For ZnO, Bamiduro et al. had also found a merging process after stacks of several nanoplatelets formed [24]. Samples obtained after 2 h and 5 h, respectively (see Figure S1b and S1c in Supporting Information File 1), showed no visible difference to the sample obtained after 30 min. There were still type III structures found consisting of type II particles.

**Figure 2:**
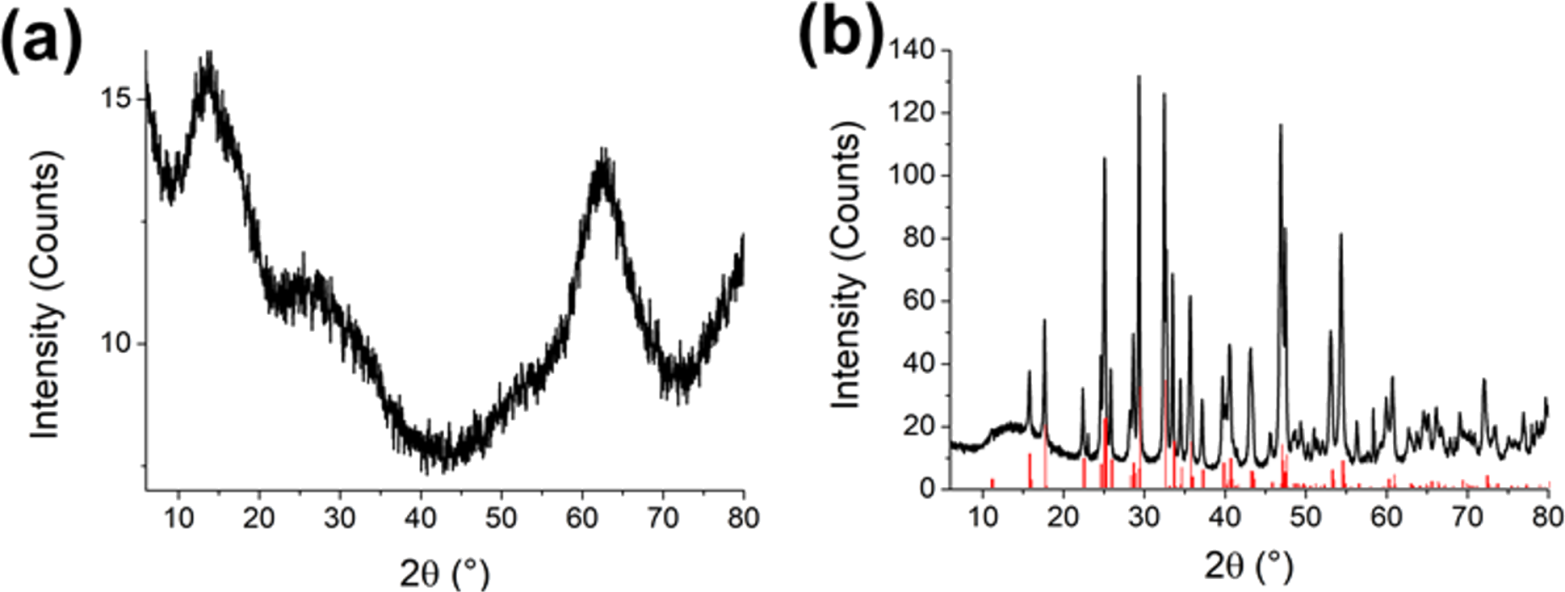
X-ray diffractograms of a sample after a reaction time of (a) 30 min and (b) 18 h (the red lines correspond to stibnite, COD 9003460).

After 8 h, the dispersion started to turn brownish in color. The amorphous, spherically shaped type II nanoparticles (see X-ray diffraction spectrometry, XRD in Figure 2 below) began to form the first crystalline, rod-shaped particles (see Figure S1d in Supporting Information File 1), which were about 1 µm in length and about the same width as the diameter of the type II nanoparticles. After 12 h, the dispersion got a darker brownish color and rod-, branch-, and urchin-like particles were found (Figure 1d). These particles were 2.6 ± 0.9 µm in length with an aspect ratio between 4 and 5 and resembled the type III structures. Still, there were mainly particles of the prior stage remaining.

**Figure 3:**
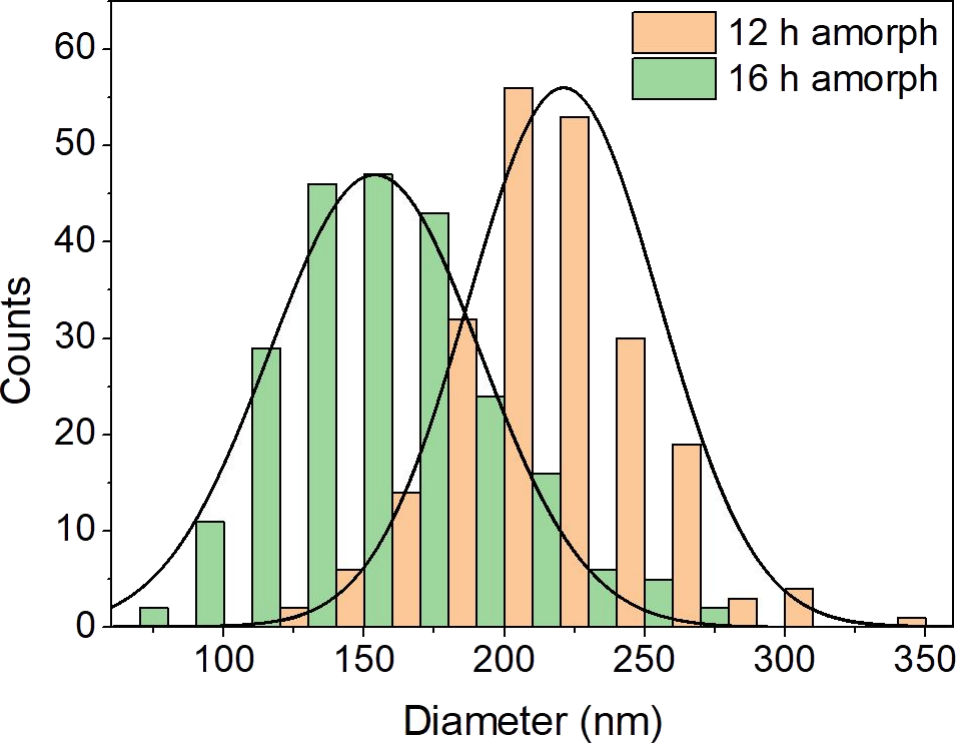
Histograms showing the size distribution of the spherical, amorphous particles in the samples obtained after 12 and 16 h, respectively. A decreasing diameter with progressing reaction time is visible and likely due to Ostwald ripening. The size distribution curves were calculated assuming a Gaussian distribution.

The crystalline rods grew apparently at the expense of the spherical, amorphous nanoparticles in an Ostwald ripening process. This assumption is supported by the decreasing diameter of the spherical, amorphous nanoparticles from 220 ± 30 nm to 150 ± 40 nm, which one can see in Figure 3, as the reaction progresses from 12 h to 16 h (Figure 1e) and by the decreasing amount of these particles (see Figure S2 in Supporting Information File 1). At this stage, the rods had a length of 5.7 ± 1.8 µm. Rod-like crystal growth by the dissolution of spherical, amorphous nanoparticles had already been suggested by Validžić et al. for similar processes at a higher temperature [20], further supporting this assumption.

The rods grew anisotropically, i.e., preferentially in the longitudinal direction, as shown by the changing aspect ratio from ≈4–5 to ≈6 after 12 h and 16 h, respectively. This observation agrees with the findings of authors who had described a growth along the c-axis of Sb_2_S_3_ nanomaterial [26–27]. This anisotropic growth corresponds to the orthorhombic structure of stibnite, the crystalline form of Sb_2_S_3_ [28].

After 18 h (see Figure S1b in Supporting Information File 1), the solution got grayish-black, and no more spherical particles were found. The size of the rods obtained after 18 h was identical to that in the sample after 30 h within the measurement uncertainties (5.5 ± 1.9 µm and 5.4 ± 1.6 µm). Histograms of the length and width distribution of the crystalline particles obtained after 16 h, 18 h, and 30 h can be found in Figure S3 in Supporting Information File 1.

TEM images of growing rods show layered structures (see Figure S4 in Supporting Information File 1) with bristle-shaped tips, which got more distinct with increasing reaction time (see Figure S5 in Supporting Information File 1). The bristles became thicker but did not appear to change in length at reaction time points between 16 h to 30 h. These findings suggest a fiber-like growth at the tips and a layered growth around the individual bristles and the whole rod. However, the particles did not seem to be bundles of nanowires, leading to the assumption that the different, fiber-like growing parts of one particle were fusing. Since the tips remained the bristle-like shape, even after the growth stopped after 18 h reaction time, a merging process was excluded. The growth of individual particle fragments, such as bristles, has been described in the literature as a dendrite-like splitting or branching of primary particles in an autoclave synthesis with ethylene glycol or polyethylene glycol as solvent [29–30]. The authors reasoned the cleavage at the particle tips by weak van der Waals forces between (Sb_4_S_6_)*_n_* chains, of which the particles consisted, or by strongly bound ligands interfering with the crystal growth, respectively. However, as crystal growth is a kinetically controlled process, mild reaction conditions lead to a delayed growth in the preferred direction, and the other crystal planes also grew. Hence, it is likely that integration of the dissolving amorphous particles would fuse the fibers of the crystalline ones. This behavior has also been found by Validžić et al. in a different approach of synthesizing Sb_2_S_3_ nanoparticles at a higher temperature (240 °C) [31].

Table 1 gives an overview of the characteristics of the particles received after different reaction time points. One can see the size of the amorphous nanoparticles/aggregates, the size and aspect ratios of the crystalline particles, the corresponding molar ratio of Sb and S obtained by EDX, and the associated band gap obtained by reflectance measurements (see discussion of the optical data below).

XRD measurements of an orange-red (30 min) and a grayish-black (18 h) sample were exemplarily performed to examine the structure of the samples. The diffractograms shown in Figure 2 revealed a low crystallinity for the sample obtained after 30 min (Figure 2a) as no specific diffraction peaks were found, and a high crystallinity in accordance with the stibnite structure (COD 9003460) for the sample was obtained after 18 h (Figure 2b). These results show that the rather spherically shaped orange nanoparticles were mainly amorphous and crystallized into rod-like, grayish-black particles.

Thus, the kinetics of the reaction progress could be followed by the dispersion color. As long as there were only amorphous structures present, the dispersion had an orange-red appearance. We assumed that some of the amorphous, orange-red type III structures acted as crystallization nuclei after 7–9 h. The particles started to crystallize in the shape of the superordinate type III structures described previously, leading to a brownish color. Owing to the preferred growth direction, rods, branch-like, or urchin-like stibnite particles were finally received, which had a grayish-black appearance.

**Figure 4:**
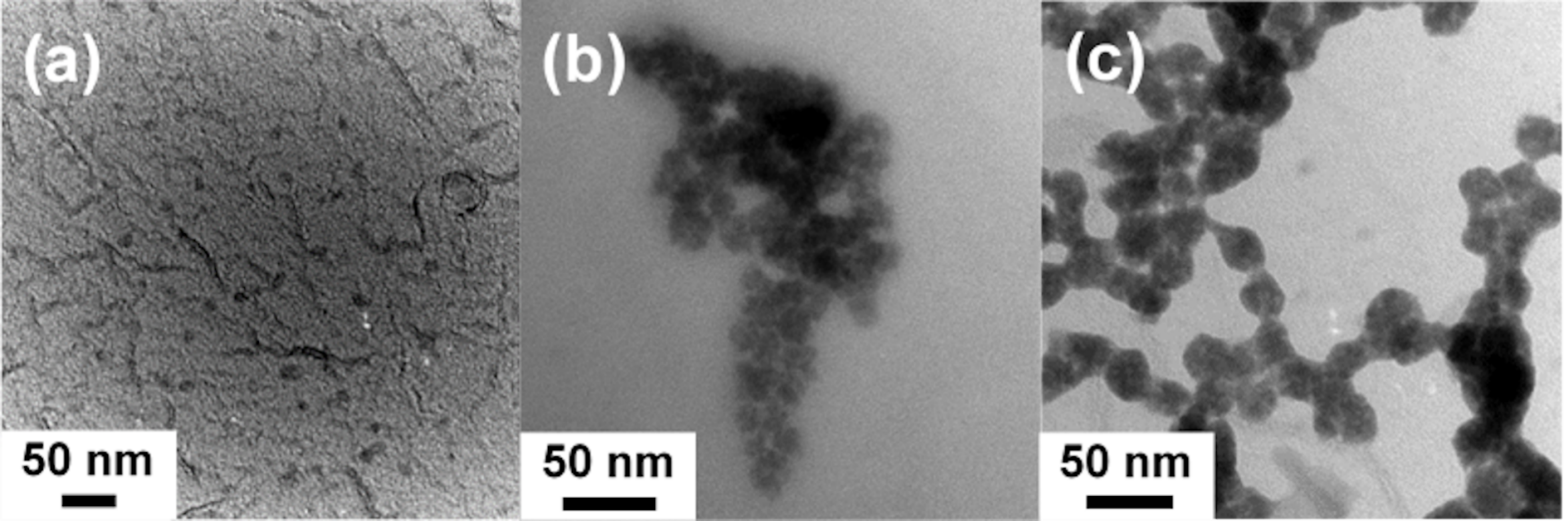
Nanoparticles obtained after 30 s reaction time: (a) small, individual nanoparticles, (b) raspberry-like, larger nanoparticles, intermediate state, and (c) raspberry-like, larger nanoparticles, final state.

A major advantage of slowing down the reaction kinetics was the possibility of looking at the early stage of the reaction. Therefore, in addition to the experiments described above, the reaction was also stopped after 30 s when the solution was still transparent. In this case, next to nanoparticles of a similar size and shape as the type I nanoparticles obtained after 2 min, an even smaller species of seed particles (type 0) could be observed (Figure 4a). These type 0 seed particles were 5–10 nm in size and seemed to assemble into the larger type I nanoparticles as the latter ones had a raspberry-like appearance (Figure 4a and Figure 4b). The larger nanoparticles had sizes between 15 and 35 nm (Figure 4b), with the particle fraction of a size around 35 nm found more frequently (Figure 4c). Together with the finding that the type I nanoparticles were also around 35 nm in diameter (see Figure 1), this led to the assumption that there was an aggregation and merging step from the type 0 to the type I nanoparticles in addition to the one occurring from the type I to the type II nanoparticles. Thus, there was a double hierarchical assembly and subsequent merging process of the amorphous nanoparticles. It was not possible to isolate visible nanoparticles immediately after injection.

**Figure 5:**
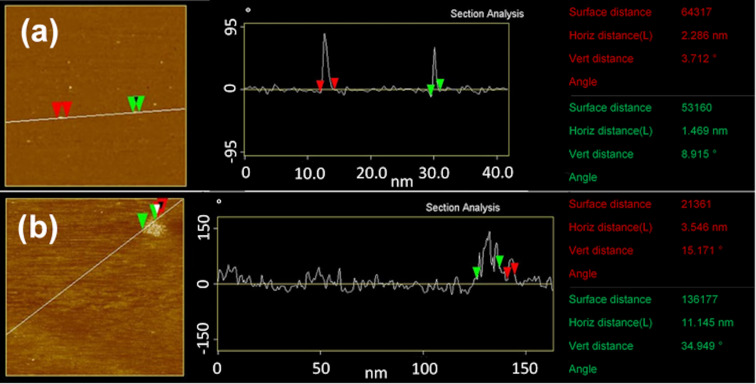
AFM measurements of nanoparticles obtained after 30 s reaction time: (a) individual single nanoparticles and (b) cluster of small nanoparticles of about the same size as the individual ones in (a). Both images were taken of the same sample. The results contain the measured area, with height differences displayed by a difference in brightness, a height evaluation along a drawn line (lever deflection in ° vs distance in nm), and a sum-up of marked distances along the drawn line. The particle/cluster size is given as the horizontal distance (Horiz distance (L)).

Atomic force microscopy (AFM) as an additional method of size determination was applied to confirm the TEM results of the sample obtained after 30 s reaction time. AFM enables imaging of the nanoparticles under milder conditions than TEM and at ambient conditions so that thermal damage of the nanostructures due to the electron beam could be excluded [32]. The data of the AFM measurements are displayed in Figure 5.

On the one hand, one can see single deflection peaks in Figure 5a, which are 1.5 nm and 2.3 nm in width (green and red marks). On the other hand, Figure 5b shows four deflection peaks directly next to each other. The width of the peaks is about 3.5 nm (red mark), and they appear rather individually. It is suggested that these single deflection peaks correspond to the type 0 nanoparticles already found in TEM (Figure 4a). The size difference to the TEM data is likely due to damage by the electron beam, which caused the particles to appear larger. Consequently, the stacked deflection peaks (Figure 5b, 14.7 nm, red and green mark) correspond to a nanoparticle cluster similar to those found in Figure 4b.

### Chemical composition

**Figure 6:**
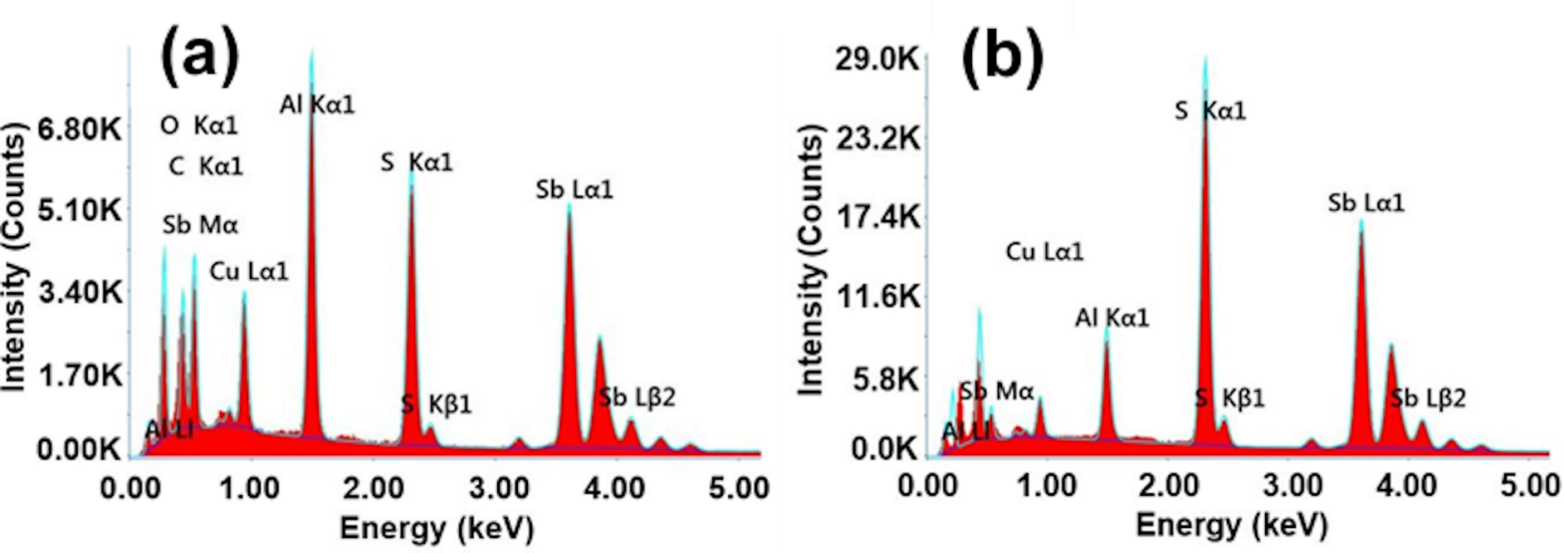
EDX spectra of the nanoparticles obtained after (a) 2 min and (b) 5 min of reaction time. The samples were measured on a carbon-coated copper grid on an aluminum holder, explaining the detection of these elements. Oxygen may have been detected due to contamination of the sample, the grid, or the holder.

To confirm the XRD results for stibnite and to examine the composition of the amorphous particles, an EDX analysis was performed in conjunction with SEM for selected samples obtained after reaction time points from 2 min to 30 h. It was not possible to examine the samples obtained after 30 s since the yield was too low at this reaction stage. The sample that had reacted for 2 min (Figure 6a) contained less sulfur than expected by the stoichiometric ratio of Sb_2_S_3_. However, the results of the samples obtained after reaction time points between 5 min and 30 h were all in good agreement with the stoichiometric ratio of antimony and sulfur in Sb_2_S_3_ (Table 1, Figure 6b, and Figures S6 and S7 in Supporting Information File 1).

The nonstoichiometric Sb/S ratio in the early reaction stage was probably due to the oleylamine used in the synthesis. Several authors have already described that hydrolysis of antimony in fatty amines can lead to the formation of Sb_2_O_3_ [33–35]. Baum et al. studied the formation of copper thioantimonate by injecting a heated (60 °C) and degassed S-OlAm precursor into a heated (200–250 °C) and degassed mixture of Cu(I)Cl, Sb(III)Cl_3_, and OlAm. When they omitted sulfur and Cu(I)Cl in this process, they obtained cubic α-Sb_2_O_3_ (senarmontite) [33]. In contrast, they could synthesize the desired copper thioantimonate when they used Sb_2_O_3_ instead of Sb(III)Cl_3_ in a following synthesis. They concluded that Sb_2_S_3_ works as an intermediate product rather than as a byproduct.

**Figure 7:**
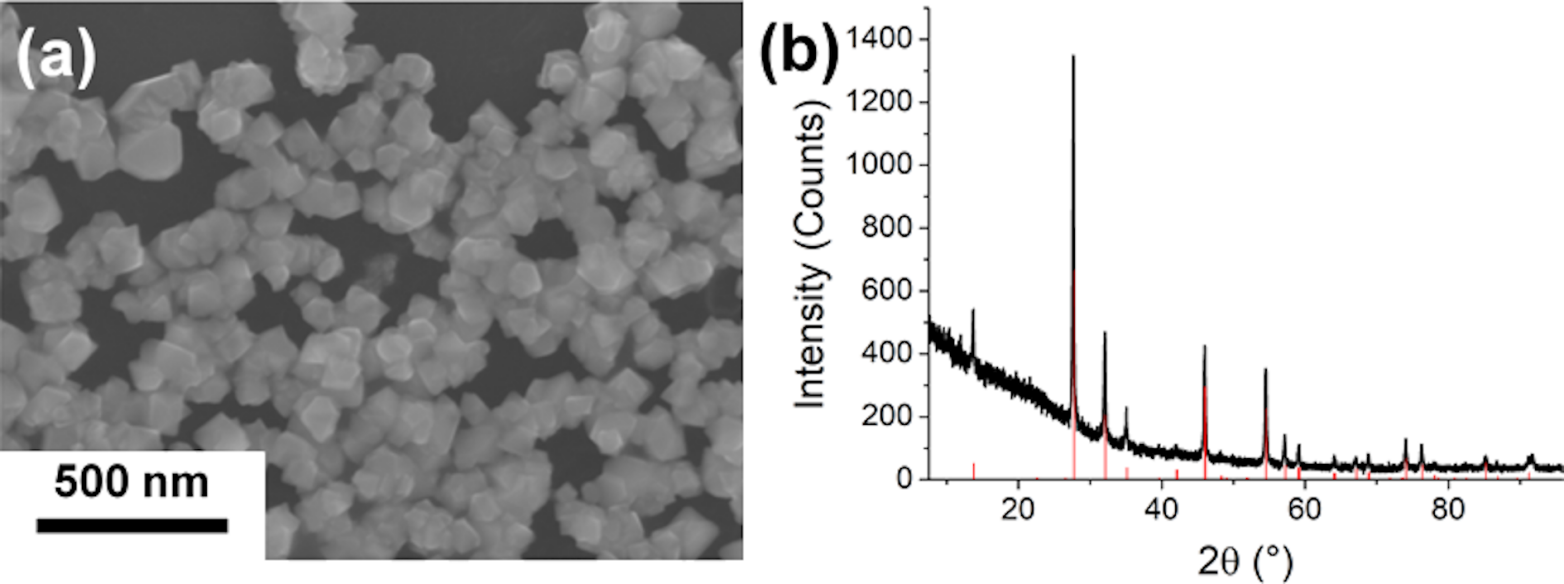
Measurement results of the white product obtained by the direct injection of the Sb precursor into oleylamine at 150 °C without the addition of sulfur: (a) SEM image and (b) XRD pattern (the red lines are corresponding to the diffraction peaks of senarmontite, COD 1011201).

To show that antimony oxide can also be formed under the reaction conditions used in this work, the Sb precursor was injected directly into the oleylamine at 150 °C without adding sulfur, and the solution turned white immediately. Figure 7 shows the SEM and XRD results of the white product obtained by this reaction. The yielded nanoparticles were 60 ± 15 nm in diameter and could be assigned to the cubic α-phase of Sb_2_O_3_, senarmontite (COD 1011201).

These results indicate that the first species formed consisted of a compound of antimony, sulfur, and oxygen, with the oxygen being replaced by sulfur with increasing reaction time while the nanoparticles transform into pure Sb_2_S_3_. This species could act as an intermediate for the particles formed at later stages or as an intermediate species formed parallel to the main reaction. It is also possible that, initially, a species that contained antimony, sulfur, and carbon residues from the precursors formed, as it has been found in high-temperature seeding processes from other metalloorganic syntheses [36]. A changing chemical composition could also be a reason for the nanoparticles to undergo a second hierarchical assembly. A similar behavior was found by Liu et al., who synthesized cobalt particles with a cobalt alkoxide intermediate [25].

**Figure 8:**
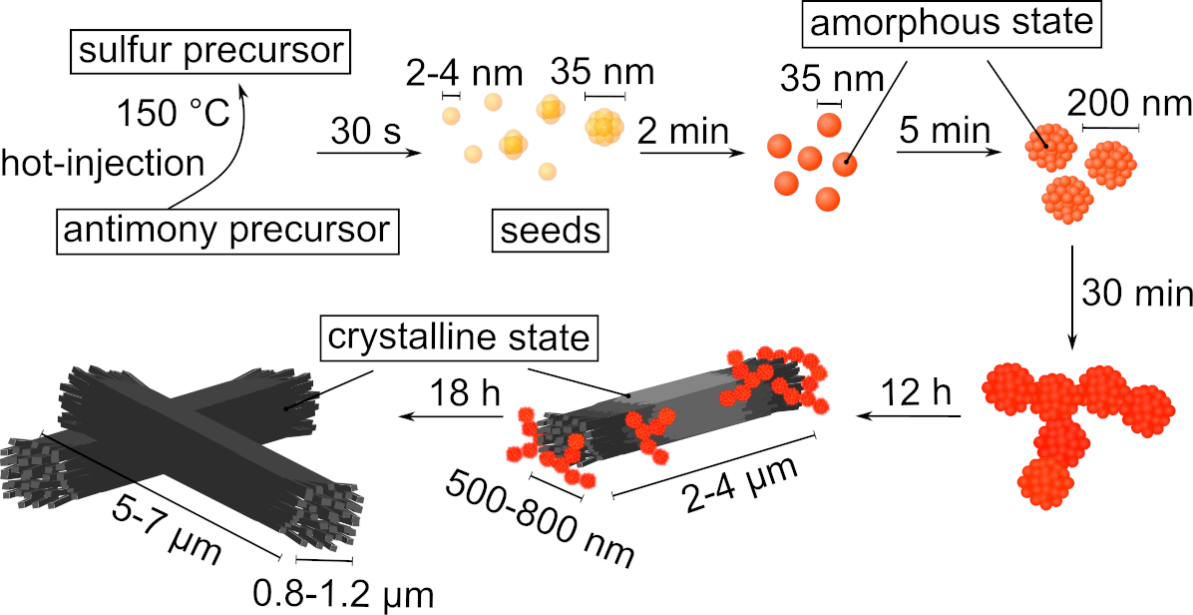
Growth scheme of Sb_2_S_3_. After injection, type 0 seeds (yellow) were formed, which turned into small type I amorphous nanoparticles (orange). These small particles aggregated and merged into type II nanoparticles (red), which assembled into superordinate type III structures before crystallizing. The crystals (black) grew at the expense of the amorphous nanoparticles in an Ostwald ripening process.

Figure 8 summarizes the results discussed above and suggests a growth mechanism for Sb_2_S_3_. The color changes during the reaction can be seen in Figure S8 in Supporting Information File 1. Instantly after injecting the colorless Sb-EHA precursor into the clear yellowish S-OlAm precursor solution at 150 °C, the reaction mixture turned orange before it turned red about 30 s later but stayed clear. At this stage, type 0 seed particles of a size of 2–4 nm (diameter determined by AFM) were formed, which assembled into 20–40 nm-sized clusters. These particles were amorphous and did not have a stoichiometric ratio corresponding to Sb_2_S_3_. The more particles assembled into clusters, the more turbid and orange the mixture became. When 2 min had passed, the mixture was completely turbid and changed to an orange color. The clustered type 0 seeds merged into type I nanoparticles of about 35 nm in diameter, which began to aggregate again into spherical structures of about 200 nm in size. At this point, the chemical composition of the particles complied with Sb_2_S_3_. The color became darker and started to turn red (≈20 min) since the aggregates merged into type II particles, which seemed to assemble into the superordinate type III structures. After 7–9 h, the solution became brownish. Most likely, this was the point at which significant crystallization began as some of the amorphous type III structures acted as crystallization nuclei. The orthorhombic crystals grew, probably at the expense of the amorphous particles, until a grayish-black mixture of crystalline material without spherical, amorphous particles (≈18 h) was finally obtained.

### Optical characterization

The optical properties of the materials were measured by reflectance spectroscopy and analyzed by applying the Tauc plot to receive the band gap values of the material [37–38]. As shown in Equation 1, the absorption coefficient α is expressed by the Planck constant *h*, the photon frequency *ν*, a constant *B*, which Davis and Mott described as the magnitude of the optical absorption constant [38], and a transition factor γ:

[1] (αhν)1γ=B(hν−Eg)

The transition factor γ depends on the type of the band gap transition. It equals 1/2 for a direct allowed transition and 2 for an indirect allowed transition.

For reflectance data, α is expressed by the Kubelka–Munk function *F*(*R_∞_*) (Equation 2), which is the quotient of the absorption coefficient *k* and the scattering coefficient *s,* which, in turn, is correlated to the reflectance of an infinitely thick specimen *R_∞_* [39]:

[2] $$F\left(R_{\infty }\right)=\frac{k}{s}=\frac{{\left(1-R_{\infty }\right)}^{2}}{2R_{\infty }}$$

In the literature, there are different opinions regarding the type of electron transition of Sb_2_S_3_. Some authors assumed a direct transition for the amorphous and the crystalline material [30,40–41], while others proposed an indirect transition [42–45].

However, amorphous materials exhibit neither an indirect nor a direct transition as these materials are highly disordered and do not have a band structure based on the Bloch theorem. Nevertheless, the electronic states in amorphous materials can be divided into localized and delocalized states, forming a so-called mobility gap [46]. Initially, the Tauc plot (Equation 1) was used to calculate band gap values for amorphous materials, i.e., mobility gaps, with a transition factor γ equal to 2 [37]. Hence, an amorphous material can mathematically be treated as a material with an indirect allowed transition.

For crystalline Sb_2_S_3_, Filip et al. [47] and Vadapoo et al. [48] did first-principle calculations of the band structures. Both found indirect transitions as energetically most favorable but with only a little difference to a direct transition. They concluded that the direct transition would most likely be dominant, especially at ambient temperature. Filip et al. defined the band gap as an "effectively direct gap". Therefore, and because most references assume a direct transition, the transition will be considered a direct one in the present work. In contrast, Validžić et al. performed calculations based on the density functional theory and found a direct band gap [31].

**Figure 9:**
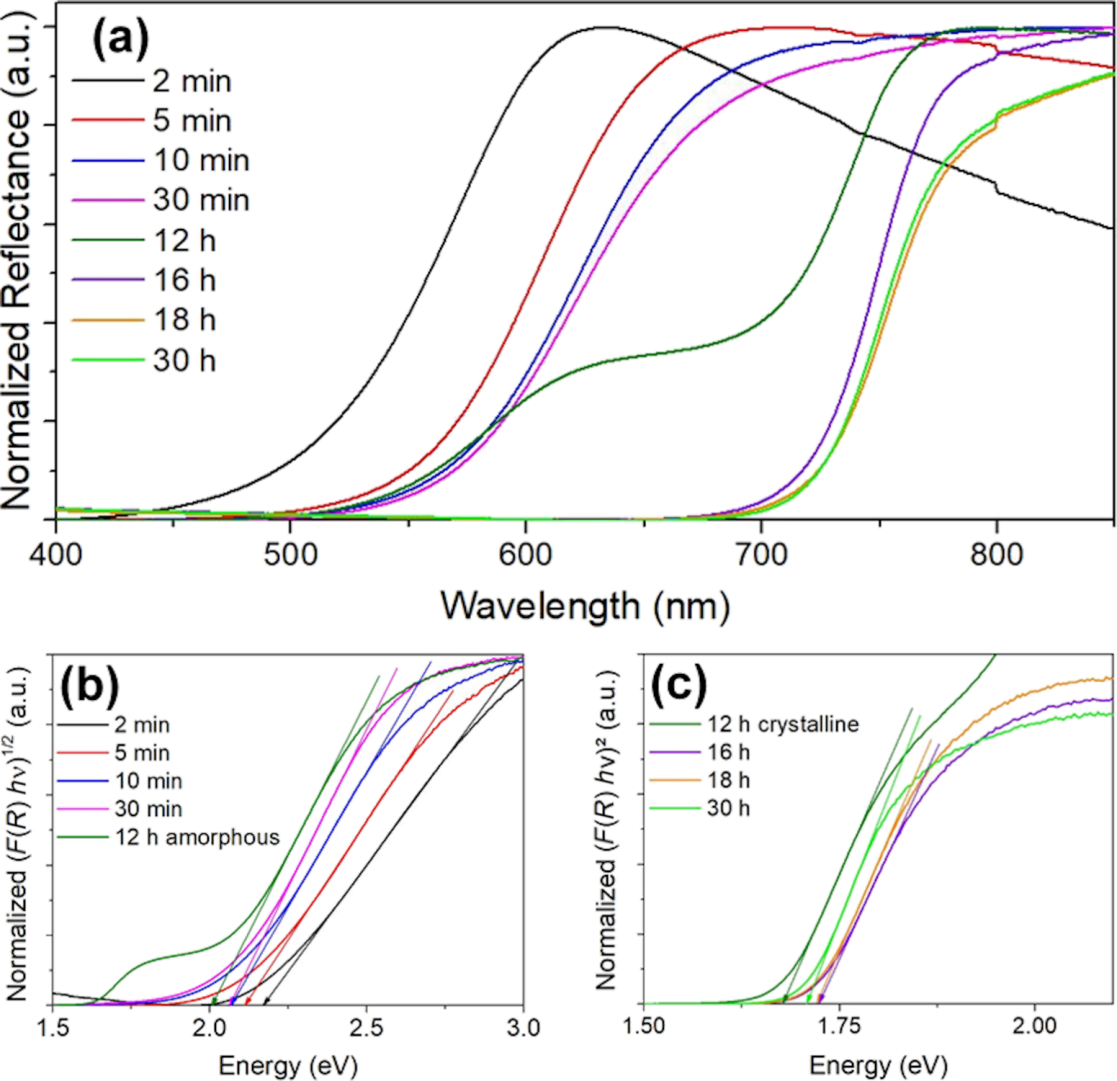
Optical characterization of Sb_2_S_3_ samples obtained after different reaction times: (a) reflectance spectra, (b) Tauc plots of the spectra of the amorphous particles, and (c) Tauc plots of the spectra of the crystalline particles. All spectra were normalized to the maximum intensity. The band transitions of the amorphous particles were treated as allowed, indirect transitions, while the transitions of the crystalline material were considered to be allowed, direct transitions. Tangents were drawn at the slope of each graph to estimate the band gap value. The sample obtained after 12 h reaction time exhibited two slopes that corresponded to the absorption of amorphous (λ < 670 nm) and crystalline (λ > 670 nm) particles present.

The optical data can be seen in Figure 9 and in Figure S9 in Supporting Information File 1. As the spectra of the 2 h, 5 h, and 8 h samples are mainly overlapping with those obtained in the other measurements, they were moved to Supporting Information File 1.

The measured reflectance data can be seen in Figure 9a. They show that the onsets of the spectra of the different samples shifted towards higher wavelengths with increasing reaction time. While the absorption of the amorphous samples approximated to a certain value (point of inflection at ≈620 nm), the absorption of the material immediately changed after crystallization and did not seem to shift significantly any more (point of inflection ≈750 nm). The spectrum obtained after 12 h reaction time exhibits two slopes at λ < 670 nm and λ > 670 nm, as well as that of the sample after 8 h reaction time (see Figure S8a in Supporting Information File 1), likely due to the simultaneous presence of amorphous and crystalline particles. Although SEM images indicated (see Figure 1e) that the sample obtained after 16 h still contained some amorphous particles, the influence of these on the optical behavior seems negligible since the second slope is no more visible.

Figure 9b and 9c show the Tauc plots of the amorphous and crystalline samples. The two slopes of the sample obtained after 12 h reaction time were fitted individually as indirect and direct transition, assuming that the first slope (λ < 670 nm) corresponds to the amorphous and the second slope (λ > 670 nm) to the crystalline particles. As one can see, the band gap values changed throughout the different samples (see Table 1 for the values) and depended on the reaction time and crystallinity of the sample. While the particles after 2 min reaction time had a band gap value of 2.18 eV, this value decreased to 2.01 eV after 12 h.

Different band gap values have already been reported for amorphous Sb_2_S_3_ nanomaterials. For example, Abulikemu et al. reported a value of 2.15 eV, while Wang et al. reported 2.02 eV for nanoparticles received from a hot-injection synthesis using different solvents [17,19]. Variation in band gap values is also known to occur in other amorphous semiconductors, e.g., amorphous, hydrogenated silicon. This was explained by different preparation conditions [49]. Different formation mechanisms can lead to differences in bond lengths and angles in an amorphous material, and therefore to a different mobility gap [46]. Hence, a decreasing mobility gap suggests that an electronic relaxation process occurs after a longer reaction time. The band fluctuations and the bond lengths and angles get closer to the band and material structure of the corresponding crystalline form until crystallization itself starts.

The material showed a different band gap energy after the crystallization had started. All samples containing crystalline particles had a band gap energy of around 1.70 eV, independent from the reaction time. This value agrees well with previously reported values for crystalline Sb_2_S_3_ particles of a similar size [50].

## Conclusion

The formation mechanism of Sb_2_S_3_ nanoparticles via a hot-injection synthesis at 150 °C was revealed. In this way, we could gain a more in-depth insight into the kinetics of particle formation, while earlier studies by Abulikemu et al. and Li et al. were focusing on the temperature-dependent evolution of Sb_2_S_3_ particles. The suggested mechanism assumes that seeds (type 0 particles) were formed directly after injecting the antimony precursor into the sulfur precursor. These seeds merged into type I amorphous nanoparticles containing a smaller percentage of sulfur than the expected stoichiometric ratio of Sb and S, possibly due to oxygen being involved in the seeding process. Subsequently, the type I nanoparticles aggregated into type II nanoparticles and formed superordinated type III structures that finally crystallized in an orthorhombic crystal structure.

Furthermore, the kinetic control of the reaction enabled tuning of the optical band gap of the amorphous material in the range of 2.18 ± 0.03 to 2.01 ± 0.03 eV. In contrast, the optical band gap of the crystalline particles decreased to a value of 1.71 ± 0.03 eV and did not change any further. The reduction of the mobility gap of the amorphous states of the particles was likely due to an electronic relaxation effect with increasing reaction time.

With the knowledge provided by this study, different strategies can be developed, capable of controlling the size of the amorphous and crystalline particles on an even broader range than it has been done up to now. In this way, the customizable application of Sb_2_S_3_ nanomaterial in solar cells and other fields of electronics and optoelectronics will be enhanced.

## Experimental

All experiments were carried out using standard glass equipment. The reaction vessels were cleaned before use with nitric acid (65 vol %, VWR Chemicals) and were subsequently repeatedly rinsed with deionized water. The nanoparticles were redispersed using an ultrasonic bath (Sonorex RK512H (860 W, 35 kHz) from Bandelin). A controlled heating rate and temperature in the reaction vessel was achieved by a temperature controller (LTR 3500, Juchheim Solingen). Injections into the reaction vessel were performed with a 14-gauge cannula (*L* = 200 mm, neoLab).

### Materials

Sb(III)Cl_3_ (>99.95%), sulfur (99.98%), EHA (>99%), paraffin oil (*d* = 0.827–0.890 g/mL), OlAm (70%), and isopropyl alcohol (IPA, 99.5%) were obtained from Sigma-Aldrich. Hexane (>98%) was purchased from Alfa Aesar, chlorobenzene (>99%) from Merck KGaA, and 1,2-dichlorobenzene (>98%) from Fisher Scientific. All chemicals were used without further purification.

### Synthesis

#### Undoped Sb_2_S_3_ nanoparticles

All reaction steps were performed under an argon atmosphere. Prior to the reaction, two precursor solutions were freshly prepared. First, the sulfur precursor, an S-OlAm solution, was produced by dissolving 1.5 mmol elemental sulfur in 6 mL OlAm via sonification in an ultrasonic bath for 10 min. Afterward, 25 mL paraffin oil was added. The solution was heated to 150 °C with a heating rate of 3.3 K/min under magnetic stirring (800 rpm). Second, an Sb(III) complex solution was prepared by adding 1 mmol Sb(III)Cl_3_ to 5 mL EHA. The mixture was magnetically stirred (750 rpm) and heated to 90 °C in an oil bath. When both precursor solutions reached the desired temperatures, the Sb precursor was swiftly injected into the precursor solution, and the reaction mixture was kept under magnetic stirring (800 rpm) at 150 °C for 60 s to 30 h. To stop the reaction, the heating mantle under the reaction vessel was replaced by an ice bath, and 15 mL hexane was injected into the reaction. The received product was precipitated by adding 30 mL IPA and separated by centrifugation at 50–2500*g* for 5–20 min (depending on the reaction time; for details, see Table S1 in Supporting Information File 1). The precipitate was redispersed in 20 mL HAS [22]. Precipitation and centrifugation were repeated twice. For the second redispersion step, 20 mL hexane instead of HAS was used. Finally, the nanoparticles were redispersed in 20 mL IPA.

### Characterization

#### SEM

SEM images were recorded with a Hitachi SU 5000 scanning electron microscope in SE mode with an electron acceleration voltage of 15 kV and a spot intensity of 40. The working distance was 3 mm. A droplet of a dispersion (*c* = 1.5–2 g/L) of the particles in IPA was dried on a carbon-coated copper grid (carbon-coating type A, 6–10 nm thickness, Cu 200 mesh, Plano GmbH). The software FIJI was used to evaluate the particle size for 200–300 particles per synthesis on several images [51].

#### TEM

A Zeiss EM 109 was used at 80 kV acceleration voltage to record the TEM images. The grid preparation and image processing were performed as stated above for SEM.

#### AFM

AFM was performed with a Multimode quadrex SPM with Nanoscope IIIe controller (Veeco Instrument Inc) operated under ambient conditions to determine the particle size using the sample in the form of a highly diluted solution. The drive frequency was kept constant during the imaging, while the drive amplitude was set to 7171 mV.

#### EDX

Elemental analysis was performed with an EDAX X-ray detector (Octane Elect Plus) attached to the SEM. The SEM was run with an acceleration voltage of 15 kV and a spot intensity of 50. The working distance was 10 mm. The resolution of the detector was 126.2 eV.

#### Reflectance measurements

Reflectance measurements were performed with a Cary 5000 UV–vis–NIR spectrometer (Agilent Technologies) equipped with an integrating sphere (internal DRA 2500). Particles were measured as a dispersion in IPA (*c* = 2–2.5 g/L) in standard cuvettes made of special optical glass (OS, Hellma) in the range of 400 to 850 nm. At 800 nm, the detector of the instrument changed, which caused a small artifact at this wavelength. While this artifact is visible in Figure 7a, it becomes negligible in Figure 7b and 7c because it corresponds to an energy of 1.55 eV, which is not in a relevant range for the band gap analysis of the measured samples, and the intensity decreases to an invisible level. The estimation of the accuracy of the Tauc method was based on a study by Viezbicke et al., who evaluated the accuracy of the Tauc plot for 120 individual analyses of polycrystalline ZnO and found a deviation of about 0.03 eV [52].

#### XRD

For XRD measurements, a minimum amount of 10 mg of dried particles was used. The samples were measured in a capillary in transmission geometry. Two different diffractometers were used to perform the measurements. The first XRD device was a Bruker D8 Advanced equipped with a LYNXEYE XE-T detector and a Cu Kα_1_ radiation source (40 kV, 40 mA) with a radiation wavelength of 0.15405 nm. The angle range of the measurements was 6–80° 2θ with a step size of 0.025°. The second device was a STOE STADI P equipped with a Dectris MYTHEN2 R detector and a Cu Kα_1_ radiation source (40 kV, 40 mA) with a radiation wavelength of 0.15405 nm. The angle range of the measurements was 6–96° 2θ with a step size of 0.015°.

## Supporting Information

File 1 Additional SEM and TEM images, EDX data, and synthesis details.
