# Supplementary material for: Revealing the formation mechanism and band gap tuning of Sb2S3 nanoparticles
Source: Beilstein J Nanotechnol. 2021 Sep 10;12:1021–33. doi: 10.3762/bjnano.12.76 (PMC8450970; doi:10.3762/bjnano.12.76)
Supplement: File 1 — Additional SEM and TEM images, EDX data, and synthesis details. [file Beilstein_J_Nanotechnol-12-1021-s001.pdf]

BEILSTEIN JOURNAL OF NANOTECHNOLOGY

Supporting Information

for

Revealing the formation mechanism and band gap tuning of Sb_2S_3 nanoparticles

Maximilian Joschko, Franck Yvan Fotue Wafo, Christina Malsi, Danilo Kisić,
Ivana Validžić and Christina Graf

Beilstein J. Nanotechnol. **2021,** *12,* 1021–1033. doi:10.3762/bjnano.12.76

Additional SEM and TEM images, EDX data, and synthesis details

License and Terms: This is a supporting information file under the terms of the Creative Commons Attribution License (https://creativecommons.org/licenses/by/4.0). Please note that the reuse, redistribution and reproduction in particular requires that the author(s) and source are credited and that individual graphics may be subject to special legal provisions.
The license is subject to the *Beilstein Journal of Nanotechnology* terms and conditions: (https://www.beilstein-journals.org/bjnano/terms)

Content:

1. Additional electron microscopy images

2. EDX data supporting the chemical composition

3. Additional synthesis information

4. Additional optical data

1. Additional electron microscopy images

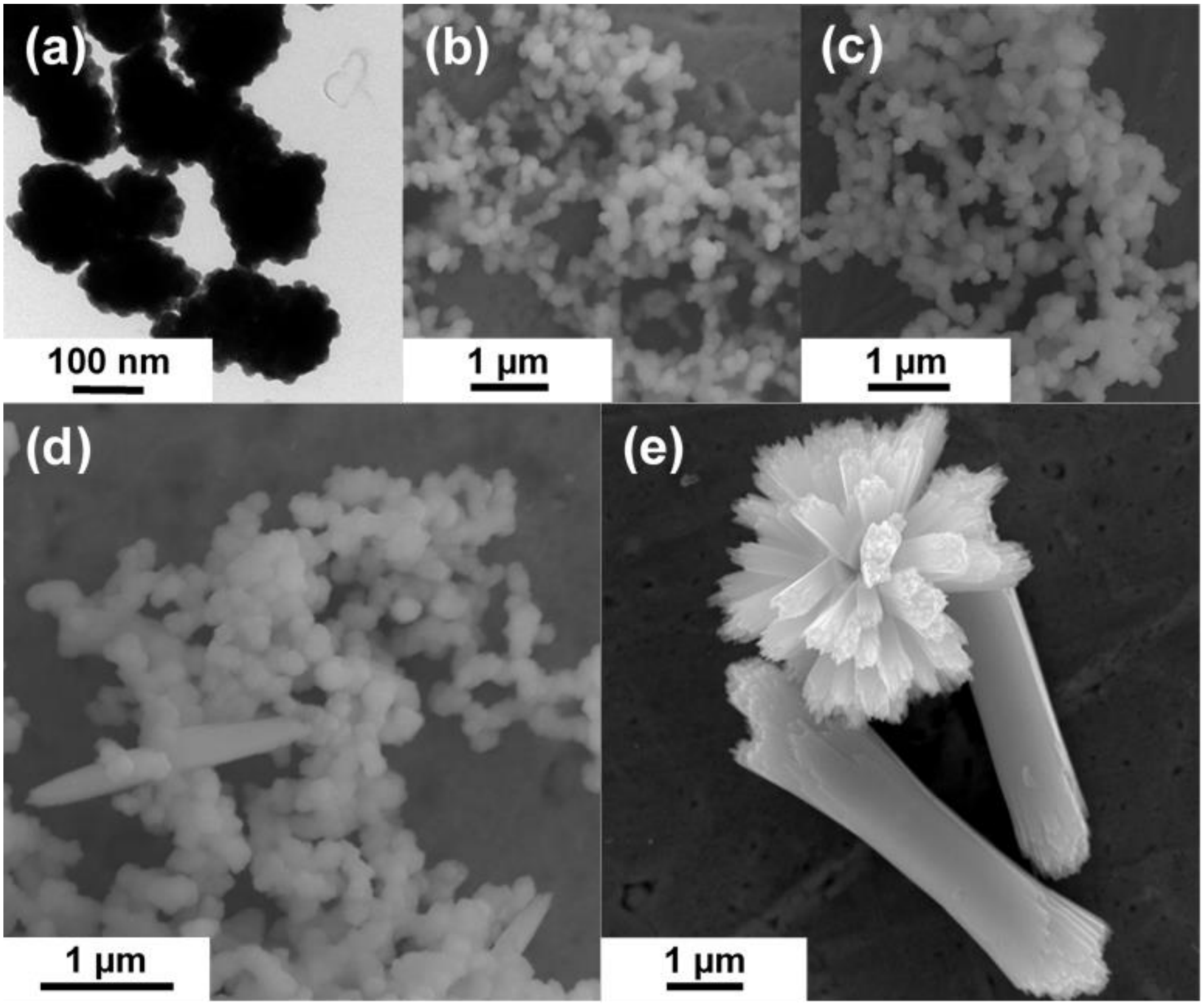

Figure S1: EM images of Sb_2S_3 nanoparticles received after different reaction times: (a) 10 min (TEM), (b) 2 h (SEM), (c) 5 h (SEM), (d) 8 h (SEM), and (e) 18 h (SEM).

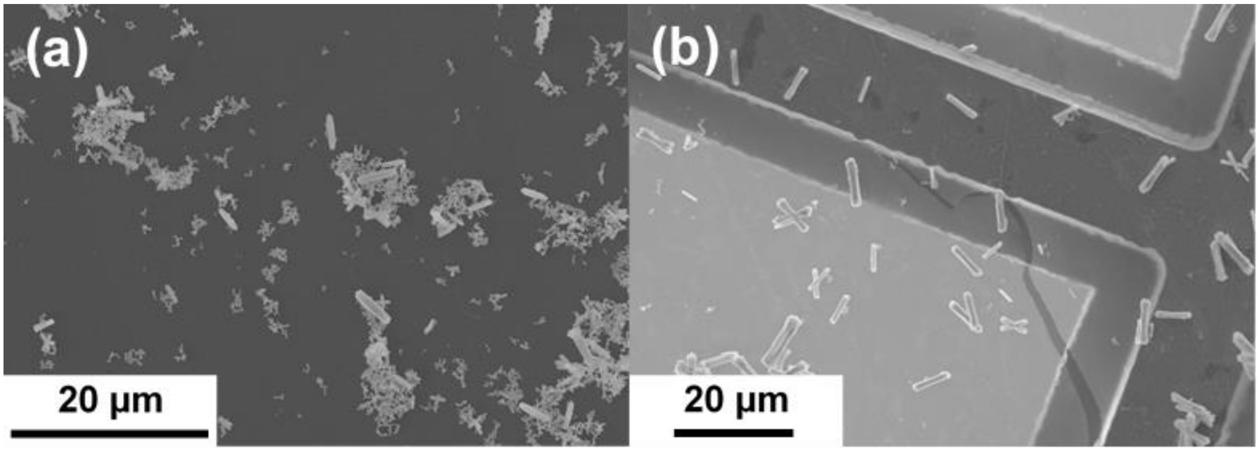

Figure S2: SEM images of Sb_2S_3 nanoparticles received after different reaction times: (a) 12 h and (b) 16 h. While there are a lot of small, spherical particles and little rods visible after 12 h, this is vice versa after 16 h.

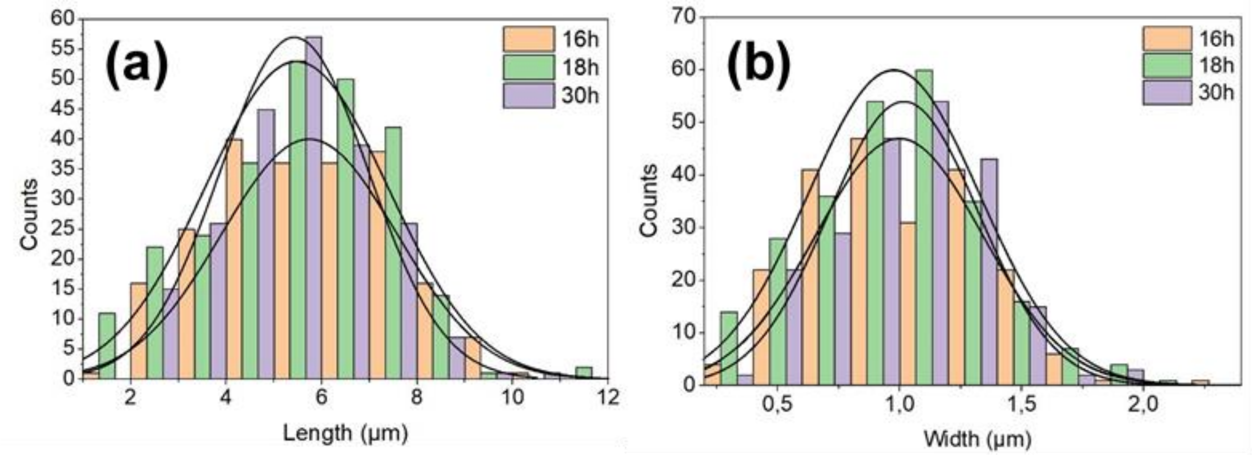

Figure S3: Histograms of the size distribution for the particles obtained after 16, 18, and 30 h for (a) length and (b) width, respectively. The size distribution curves were calculated assuming a Gaussian distribution.

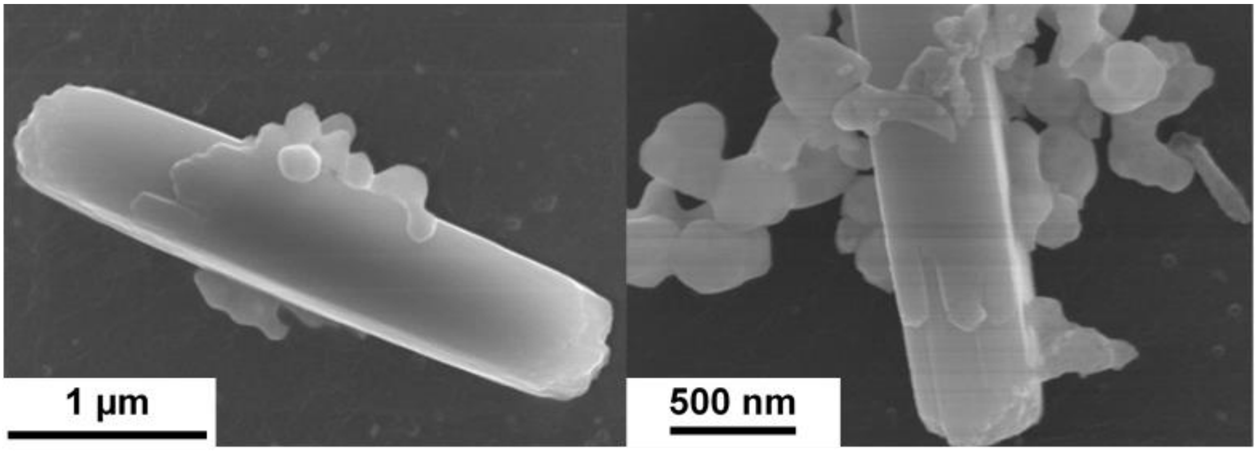

Figure S4: EM images of Sb_2S_3 nanoparticles: Layered growth after 12 h reaction time.

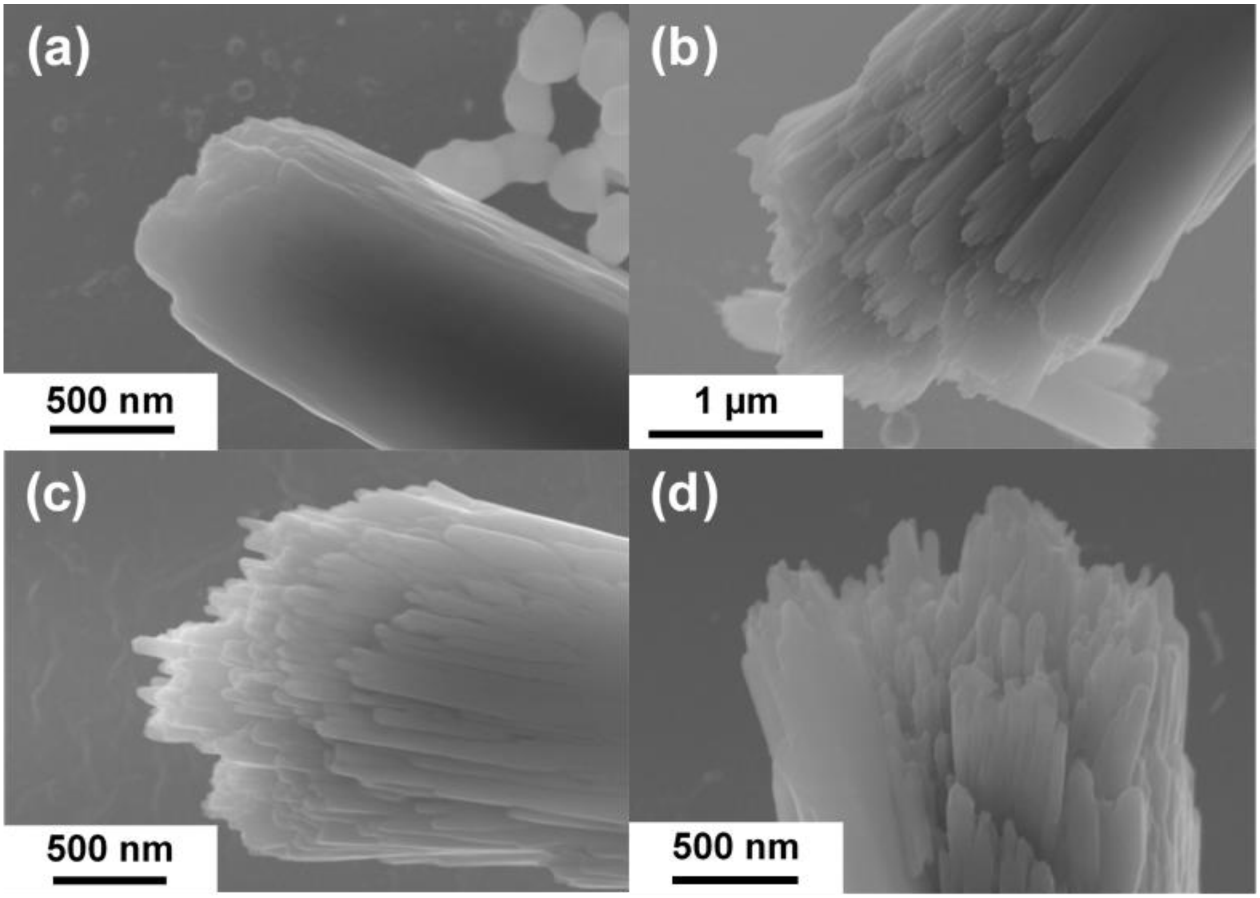

Figure S5: EM images of Sb_2S_3 nanoparticles: bristle-like tips after (a) 12 h, (b) 16 h (tip width: 40 ± 15 nm), (c) 18 h (tip width: 70 ± 15 nm), and (d) 30 h (top width: 80 ± 20 nm).

2. EDX data supporting the chemical composition

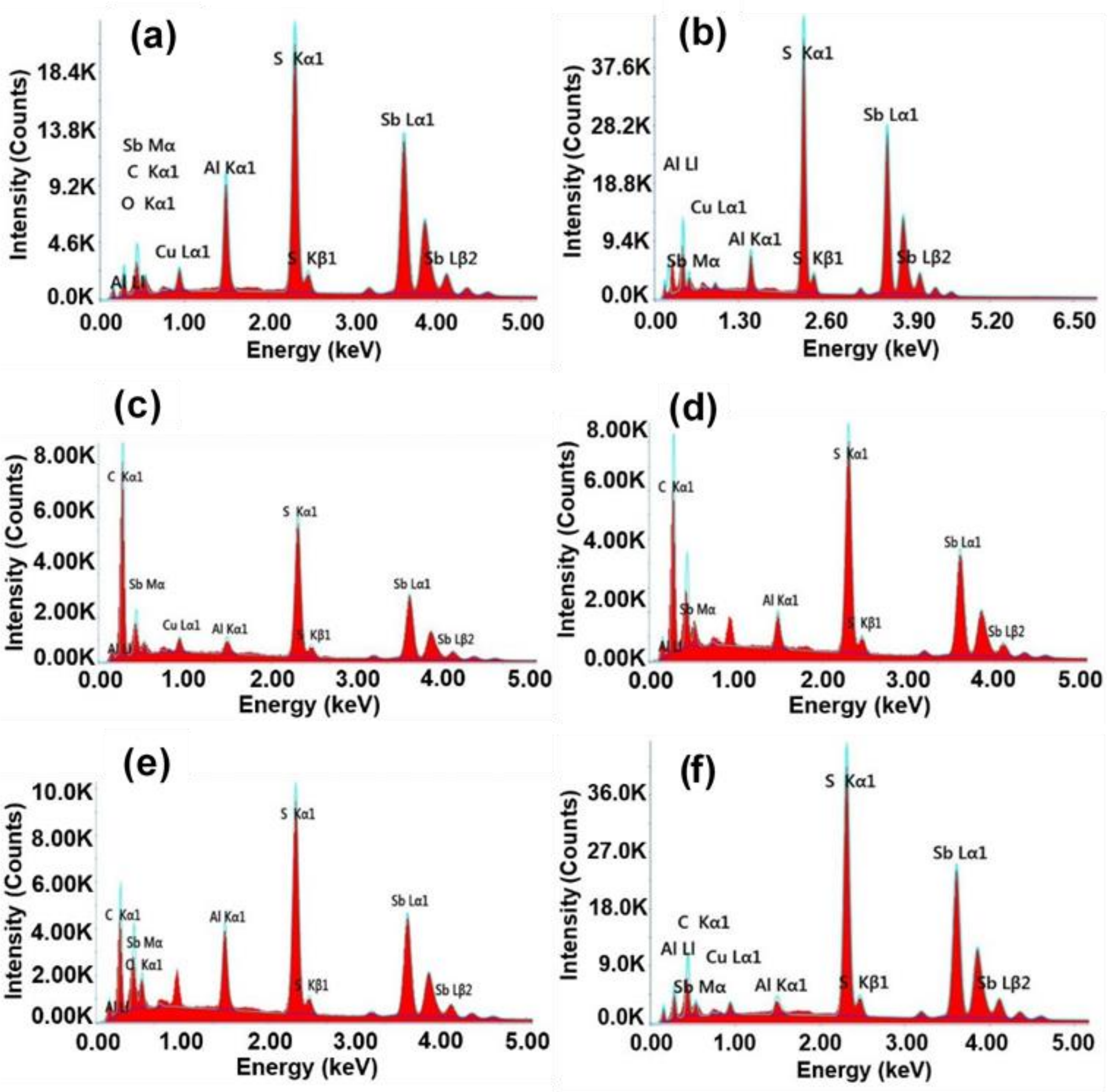

Figure S6: EDX data of Sb_2S_3 samples with amorphous particles as the only or main particle fraction after different reaction times: (a) 10 min, (b) 30 min, (c) 2 h, (d) 5 h, (e) 8 h, and (f) 12 h.

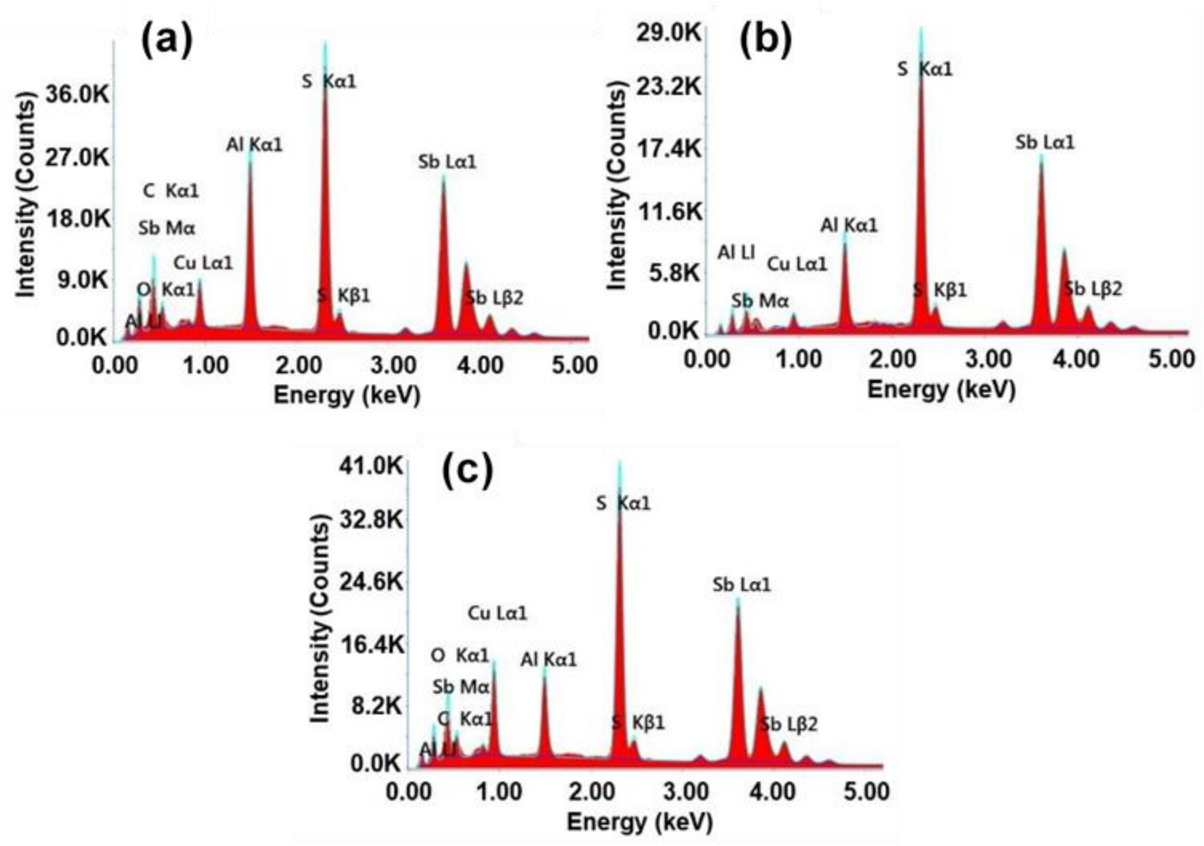

Figure S7: EDX data of Sb_2S_3 samples with crystalline particles as the only or main particle fraction after different reaction times: (a) 16 h, (b) 18 h, and (c) 30 h.

The samples were measured on carbon-coated copper grids placed on an aluminum holder, so these elements are found in all samples. Traces of oxygen may have been detected due to contamination or slight oxidation of the grid or the holder. The atomic ratios of Sb/S are given in Table 1.

3. Additional synthesis information

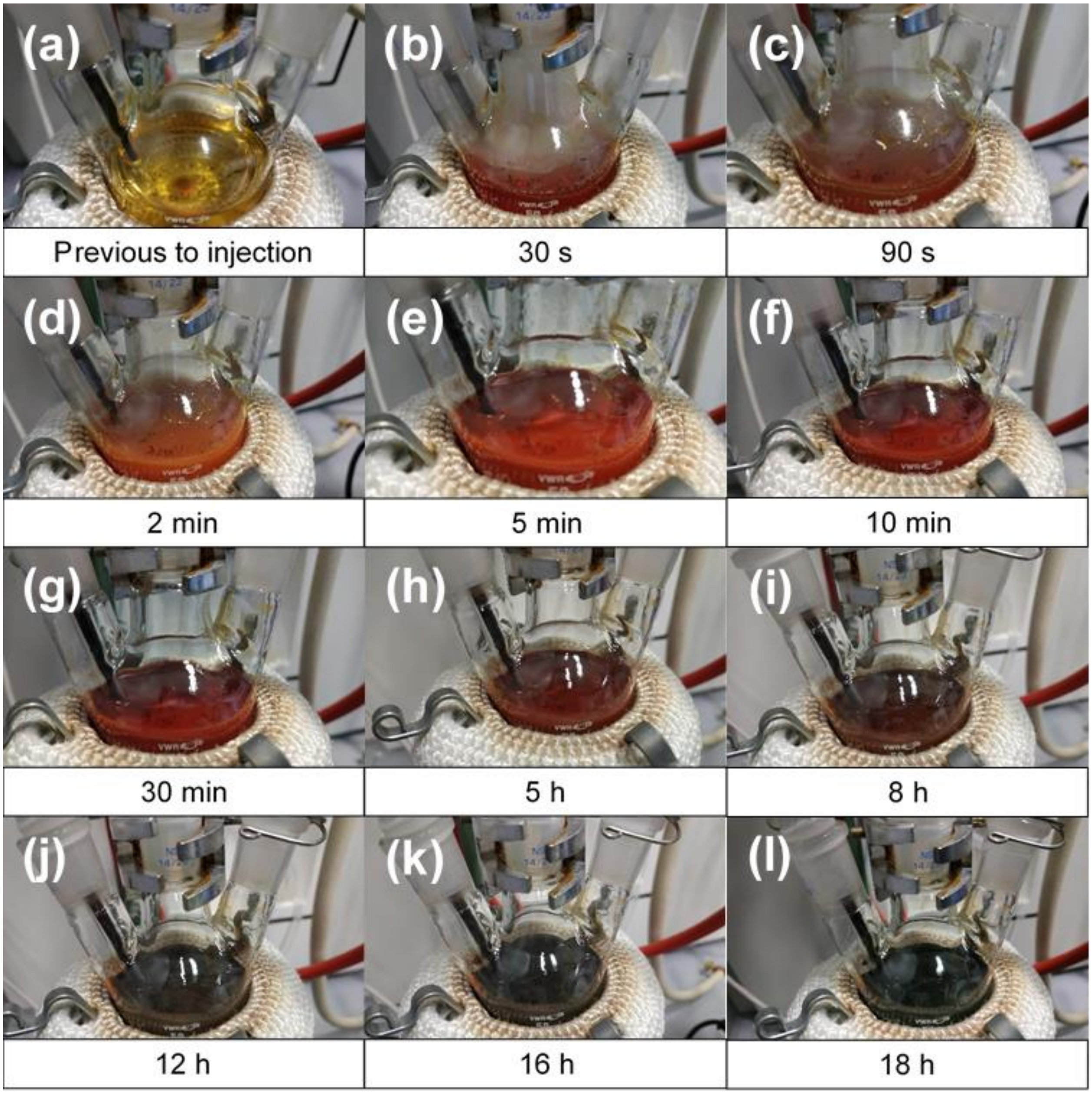

Figure S8: Color changes of Sb_2S_3 samples after different reaction times: (a) previous to injection, (b) 30 s, (c) 90 s, (d) 2 min, (e) 5 min, (f) 10 min, (g) 30 min, (h) 5 h, (i) 8 h, (j) 12 h, (k) 16 h, and (l) 18 h.

Table S1: Centrifugation speed and duration of the three centrifugation steps for the samples obtained after different reaction times.

**Reaction time**	**Step 1**		**Step 2**		**Step 3**	
	Acceleration (g)	Duration (min)	Acceleration (g)	Duration (min)	Acceleration (g)	Duration (min)
2 min	150	5	250	10	2500	20
5 min	100	5	250	5	2500	10
10 min	100	5	250	5	2500	10
30 min	100	5	150	5	1000	10
**2 h**	100	5	150	5	1000	10
5 h	100	5	150	5	1000	10
8 h	100	5	150	5	1000	10
12 h	50	5	100	5	500	5
16 h	50	5	100	5	500	5
18 h	50	5	100	5	500	5
30 h	50	5	100	5	500	5

For the centrifugation, 20 mL dispersion was filled in a 50 mL centrifugal tube. Subsequently, 30 mL isopropyl alcohol was added to precipitate the particles. Afterward, the mixture was centrifuged. The centrifugation speed was adjusted according to the increasing particle size.

4. Additional optical data

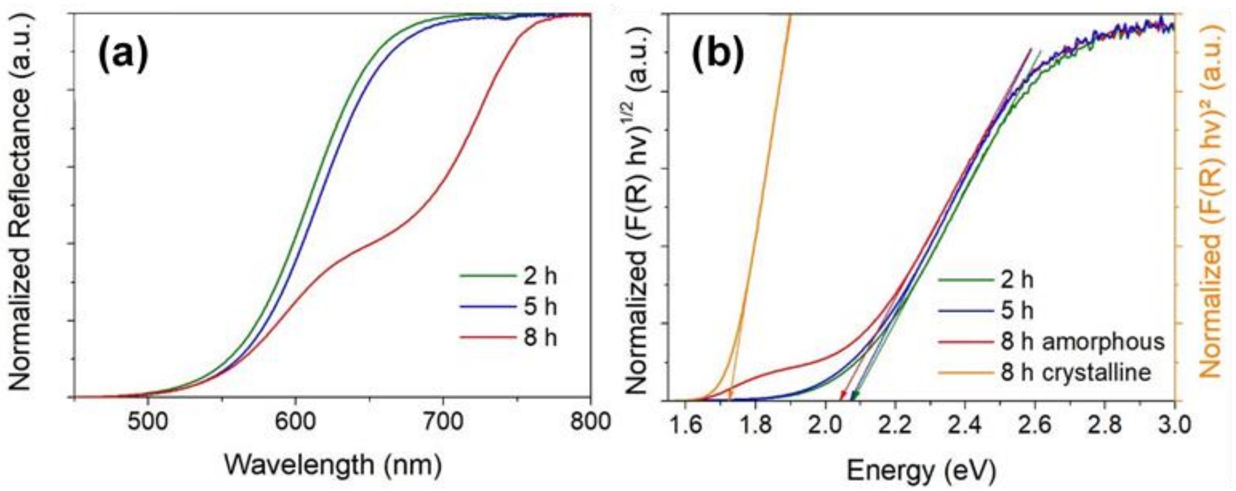

Figure S9: Optical characterization of Sb_2S_3 samples obtained after different reaction times: (a) reflectance spectra and (b) corresponding Tauc plots. All spectra were normalized to the maximum intensity. The band transitions of the amorphous particles (2 h, 5 h, and 8 h amorphous) are treated as allowed, indirect transitions, while the transition of the crystalline material (8 h crystalline) is considered to be an allowed, direct transition. Tangents were drawn at the slope of each graph to estimate the band gap value. The sample obtained after 8 h reaction time exhibits two slopes which correspond to the absorption of amorphous ($\lambda < 670$ nm) and crystalline ($\lambda > 670$ nm) particles present.
